# Notes and News

**Published:** 1892-08-27

**Authors:** 


### Notes and News.

COLLECTED AND COMMUNICATED.

There are great rejoicings in Cork at Sir John Arnott's generous gift of £1,000 to the city hospitals. A few months ago, at Lady Zetland's request, Lady Arnott started a collection in aid of the establishment of a national hospital for consumptives in Ireland, and the Cork people responded in the shape of a sum of £1,150. Sir John Arnott, knowing that this large sum goes out of Cork, and knowing that the city hospitals are much in need of funds, has given the £1,000 in compliment to Cork liberality.

The depressed state of trade seems to be affecting a good number of our institutions. Among those complaining of a diminution in the funds is the Ingham Infirmary, South Shields. This infirmary being in the centre of the district, which has lately been going through troublous times, has naturally suffered to a serious extent. But the allegations as to want of prompt attention to surgical cases, made at the annual meeting by Mr. Peter Thornton and some others, will, perhaps, have had some influence in the direction of diminishing receipts from the miners. The piano and the tennis-ground, according to these allegations have an undue fascination for the doctors, but it is to be hoped, for the credit of the institution, that complaints have been unduly exaggerated.

The plans of the new Infirmary at Southport have now been decided on, and those designed by Mr. C. Sydney Ingham have been accepted. The architecture of the building is to be a free treatment of the Flemish. The central portion of the main frontage will be two storeys in height, and the ends one. There will be bed-rooms for the accommodation of sixteen nurses, and at each end of the main building a ward will be provided for twenty patients, in addition to four separation wards. There is to be a large day-room, ward kitchen, nurses' sitting-room, sitting-room for patients, and an operation-room with a north light. A feature of the building is the children's ward, which is circular, with accommodation for twelve cots, and which is practically a distinct and separate hospital. When out for exercise the children will be kept apart from the adult patients.

The privilege enjoyed by the subscribers to the Royal United Hospital, Bath, of electing the honorary physicians is just now causing a correspondence in the local papers, which is more acrimonious than edifying. It is scarcely a matter of surprise that physicians of standing should hesitate to offer themselves for such a post under somewhat embarrassing circumstances. The prevailing opinion seems to be against the continuation of the privilege, and in favour of placing the appointment in the hands of the Committee.

Hull has just received a welcome and useful gift from Mr. Francis Reckitt, of Caenwood, Highgate, and Mr. James Reckitt, of Brough, who have presented the Royal Infirmary there with Withernsea Hotel and grounds as a convalescent home. Though admission to the Home is left to the judgment of the Infirmary Board, the property is given on the understanding that not only are patients to be sent from the Infirmary, but that other institutions in Hull and the East Riding, and even beyond the Riding, as well as private persons, will be admissible, provided their maintenance be defrayed by subscriptions or other payment.

Mr. G. Purcell has written to the Trustees of the South Charitable Infirmary and County Hospital, Cork, placing his resignation of the office of Treasurer in their hands, owing to his inability to agree with the changes recently made which he is persuaded "will not work for the comfort of the patients in the hospital." As Mr. Purcell has on several occasions pointed out the evil likely to result from the alterations, but without effect, he feels bound to resign. His objections seem aimed mainly at the increase in expenditure during recent years, and how strongly he feels in the matter can best be shown by the following extract from his letter: "It has been told us more than once at our board meetings 'that medical science and medical skill were advancing by leaps and bounds'; of this I am very dubious, but if it be true in any respect it is not in evidence as regards the past and present of your hospitals." It does not seem highly probable, after such a scathing denouncement, that Mr. Purcell will reconsider his decision as the Trustees hope.

The result of the voting on the question of the proposed extension of the Manchester Royal Infirmary has been duly declared, and the scheme of the Infirmary Board for an extension on the present site has been defeated. Not the most satisfactory feature in the matter is the number of trustees who neglected to express an opinion on the subject, displaying an apathy which was scarcely to be expected after so much controversy. A correspondent to the *Manchester Guardian*, who admits to having taken the unsatisfactory middle course of not voting at all, urges the Infirmary Board to bring forward a fresh scheme, advocating "a complete rebuilding on the present site, and the maintenance of a strong independent infirmary at Piccadilly." The majority of people will probably view this scheme in the light of the old scheme in a fresh garb. But it has been finally and decisively settled that any extension cannot be on the present site, and it remains for the Board of Management to face the matter in a business-like way. It is unfortunate that professional jealously should have helped to obscure the issues, but at least one difficulty has been cleared away by the decision of the poll.

The rules regulating the appointment of the medical staff at the Dewsbury and District Infirmary having caused some dissatisfaction, an alteration of them is now under the consideration of a committee appointed for the purpose. At present the medical appointments are held for ten years, and any vacancy which occurs, by death or otherwise, has to be filled up within three months. Any appointment now made will not be affected by an alteration of the rules.

The Derby Hospital for Sick Children has a Hospital Thursday Fund, which was inaugurated some three years ago amongst the working classes, on lines similar to that established some years since on behalf of the Derbyshire Infirmary. This fund has now made substantial progress, but still more interest in the movement on the part of the working men will yet, it is hoped, be evoked, so that through its means the institution may continue to receive constant and regular support.

The Belfast Board of Guardians is sorely tried in its mind over the question of who ought to pay for the holidays of the dispensary medical officers and apothecaries. By a resolution of June last it was decided to pay substitutes for these officers during their absence; this resolution no longer commends itself to some of the Board as imposing a hardship on the ratepayers, and an unavailing effort has been made to rescind the motion. Some members appear to have supported the resolution, acting under the impression that the Local Government Board would bear a portion of the expenditure, but this showed a very confiding trust in the generosity of Government departments. The question is to come up again for decision shortly.

Ireland and Scotland have both been looking forward to the establishment of a national hospital for consumptive patients on their respective soils, and at length the former country is in a fair way to accomplish the gratification of its desires. This is well, for had Scotland been first in the field there would have been yet another grievance to be borne by this unhappy but charming country. The sum of £10,000 required to be promised before September 1st, 1892, has been secured, mainly through the instrumentality of Lady Zetland, Miss Wynne, and Lady Arnott. The consumptive poor of Ireland, for whose benefit so much labour and thought have been bestowed, owe much to the warmth with which Miss Wynne started the movement and advocated the need of a project which was looked upon somewhat coldly in some quarters. In addition to the sum of money now secured or promised Lord Fitzwilliam has helped on the consummation of the scheme by presenting a site of nearly twenty acres for the hospital in a favourable spot near Newcastle, county Wicklow, which is specially suitable as being sheltered from cold wind, and being situated in a dry locality. The success which has crowned the project will, we trust, be a stimulus to those who are endeavouring to start a similar institution in Scotland. An attempt is now being made to secure a grant for this purpose from the funds of the Bellahouston bequest. The need of such an institution is undoubted, and if only a few rich people will come forward it will soon be supplied.

